# Prevalence and antibiotic resistance pattern of bacteria from sepsis suspected neonates at St. Paul’s Hospital Millennium Medical College, Addis Ababa, Ethiopia

**DOI:** 10.1186/s12887-023-04399-y

**Published:** 2023-11-18

**Authors:** Merema Sherif, Dessie Abera, Kassu Desta

**Affiliations:** 1 St Paul hospital Millennium Medical College, Addis Ababa, Ethiopia; 2 Department of Medical Laboratory Sciences, College of Health Sciences, Addis Ababa University, Addis Ababa, Ethiopia

**Keywords:** Bacteria, Neonate, Antimicrobial resistance

## Abstract

**Background:**

Neonatal sepsis is the major cause of neonatal mortality and morbidity, especially in low and middle-income countries. Continuous monitoring of pathogens and their antibiotic resistance pattern is crucial for managing neonatal sepsis. This study aimed to determine neonatal sepsis due to bacteria, antibiotic resistance patterns, associated risk factors and patient outcomes at St. Paul’s Hospital Millennium Medical College.

**Method:**

An institutional-based cross-sectional study was conducted on 400 neonates suspected of sepsis at St. Paul’s Hospital Millennium Medical College from March 2020 to July 2020. A questionnaire was used to collect socio-demographic information, clinical parameters and potential risk factors from study participants. About 2ml of blood was drawn aseptically and inoculated into Tryptone Soya Broth at the patient’s bedside. Bacterial identification was performed by using standard microbiological techniques. The disk diffusion method was used to determine the antibiotic susceptibility patterns of each isolated bacteria. Data entry and analysis were done using Statistical Package for Social Sciences (SPSS) version 20 software. Bivariate and multivariable logistic regressions were used to assess associated risk factors of neonatal sepsis. A p-value less than 0.05 was considered statically significant with a 95% confidence interval.

**Results:**

The overall prevalence of neonatal septicemia was 21% (84/400). Of these, 67 (79.8%) and 17 (20.2%) were gram-negative and gram-positive bacteria, respectively. *Klebsiella spp*, 37 (44%), *E. coli* 19 *(*21.6%) and *Coagulase negative Staphylococci* 13 (15.47%) were the leading cause of neonatal sepsis. Ciprofloxacin and amikacin were the most effective antibiotics for gram-negative and gram-positive bacteria. Multidrug resistance was observed in 84% of the bacterial isolates. Low birth weight and preterm were associated with neonatal septicemia (AOR = 49.90, 95% CI = 15.14-123.081, P = 0.002) and (AOR = 18.20, 95% CI = 6.835–27.541, P = 0.004) respectively.

**Conclusion:**

*Klebsiella spp* and *E. coli* were frequently isolated bacteria in our study. The proportion of multidrug-resistance was significantly high. Most isolated bacteria were resistant to ampicillin, ceftazidime, cefotaxime and gentamycin, which indicates the necessity of continuous evaluation of antibiotic resistance rate.

## Introduction

Neonatal sepsis is a bloodstream infection that occurs in infants less than 28 days of life [1]. It is the primary cause of morbidity and mortality among neonates, especially in resource limited countries [2]. Several literatures define neonatal sepsis as either early-onset or late-onset, depending on the symptoms occur before or after 72 h of life [3–5]. Early onset sepsis (EOS) is defined as sepsis occurs less than or equal to three days of birth, and is caused by microorganisms occur in the maternal genital tract before or at the time of birth [6]. Premature and low birth weight, infection during pregnancy, vaginal colonization with *Group B Streptococcus* (*GBS*) and lack of appropriate antenatal care are frequently reported risk factors of early-onset sepsis [7]. Based on global report, the incidence rate of early-onset sepsis is almost three times that of late-onset sepsis, and 70% of the causative etiological agents are mainly *GBs* and *E.coli* [8]. However, intrapartum antibiotic prophylaxis can reduce significantly the incidence of *GBS* in newborns [9].

Late-onset sepsis (LOS) begins after three days of life, mainly due to bacteria carried from the hospital or the society at the time of delivery [10]. It is usually associated with hospital and community acquired bacterial infections including intravenous catheterization. Most of these risk factors are preventable through early diagnosis and timely appropriate clinical management [8]. The most common etiological agents are *Staphylococcus aureus*, *Coagulase-negative Staphylococcus (CoNS)*, *E. coli* and *Klebsiella pneumoniae* [11–13]. However, the distribution of bacteria causing neonatal sepsis varies from country to country and even with the same geographical locations [14]. Globally, neonatal sepsis is the leading cause of morbidity and mortality in the first 4 weeks of life with an estimated annual death rate of 400,000 to 700,000, and accounts 15% of all neonatal deaths. Of these, around 42% of neonatal deaths occurred in the first week of life [8, 15, 16]. It is also the third most common cause of neonatal mortality [17]. In developing countries, nearly 50% of neonatal death is due to sepsis and the highest neonatal death rate has been reported from Sub-Saharan Africa with 27 deaths/1000 live births [18].

In Ethiopia, neonatal sepsis is the major newborn health problem which accounts 33% of all neonatal deaths [14]. According to 2011 and 2016 Ethiopian Demographic and Health Survey (EDHS) report, the neonatal mortality rate was significantly decreased from 37 to 29 deaths/1000 live births [19]. However, increasing death rate was reported in 2019 EDHS, 30 deaths /1000 lives [20]. According to a systematic review in Ethiopia, the neonatal sepsis was estimated at 45% with a range of 17–78% of neonatal sepsis [21]. These data indicate that the prevalence of neonatal death due to sepsis or other causes varies time to time, hence continuous assessment is essential to prevent and control morbidity. Another big challenge in neonatal life is the emergence of multidrug resistant bacteria which complicates the sepsis management, and around 30% of neonatal death is linked to antimicrobial resistance [22]. This is mainly due to frequent use of antibiotics without bacteriological and susceptibility evidence [23].

Therefore, reliable diagnosis including identification and antibiotic susceptibility is crucial to prevent and control neonatal mortality due to bacterial infection. Moreover, neonatal sepsis still continues to be the primary cause of neonatal morbidity and mortality globally as well as in Ethiopia. Therefore, the current study aimed to determine the bacterial etiology of sepsis and their antibiotic susceptibility pattern among neonates at St. Paul’s Hospital Millennium Medical College, Addis Ababa, Ethiopia.

## Materials and methods

### Study setting

This study was conducted at St. Paul’s Specialized Hospital Millennium Medical College (SPHMMC). It is located in Addis Ababa, the capital city of Ethiopia, and one of the largest teaching referral hospitals in Ethiopia. It was established in 1968 by Emperor Haile Selassie, and the medical school opened in 2007 under the name of Millennium Medical College. The hospital has around 800 beds from these 26 beds for pediatrics medical ward,16 beds for pediatrics surgical ward and 4 beds for pediatric intensive care unit (ICU) and 4 neonatal intensive care unit (NICU). It gives diagnostic and treatment services for 370,000- 500,000 patients per year, from these around 3600 were neonates.

### Study design and study period

An institutional-based cross-sectional study was conducted from March 2020 to July 2020.

### Source population

All neonates who were admitted to NICU of SPHMMC were the source population.

### Study population

All neonates with the age of 0–28 days who were admitted in the neonatal ward presenting with clinical features suggestive of sepsis such as difficulty in breathing, refusal to feed, fever, lethargy and respiratory distress were recruited for the study.

### Inclusion

All neonates born in or outside of SPHMMC during arrival or during stay int the hospital showing signs and symptoms of sepsis were included.

### Exclusion

Neonates who took antibiotics for the last two weeks during data collection were excluded in this study. This was to minimize false negative blood cultures because antibiotics may not kill all bacteria but it can decrease their number (unable to detect with blood culture). Neonates whose parents/ legal guardians were not available for interview were also excluded.

### Study variables

#### Dependent variables

Prevalence of bacterial isolates.

#### Independent variables

Age, sex, weight at birth, gestational week, medical devices, recovery or death of neonates, maternal malnutrition and chronic diseases.

### Sample size determination and Sampling technique

The sample size was determined using a single population proportion formula. Accordingly, the minimum sample size was calculated by taking the prevalence (p) of neonatal sepsis (46.6%) reported from Gondar specialized hospital, Ethiopia [24], the margin of error (5%), and 95% Confidence level (z = 1.96).

n = (Z α/2)^2^ * P (1- P)/ d^2^.

n= (1.96)^2^ *0.466(1-0.466)/0.05^2^ = 382.

The minimum sample size was 382, and non-probability sampling technique was employed using inclusion criteria till sample size fulfilled. Nevertheless, we have collected 400 samples.

### Data collection

Structured questionnaire was used to collect socio-demographic data, clinical characteristics and associated risk factors of neonatal sepsis. In addition, maternal information such as chronic disease, indwelling medical device, birth weight, nutritional status and HIV status were collected by reviewing medical records. Outcome of neonates assessed using checklists.

### Blood specimen collection

After obtaining written informed consent from parents or legal guardians, 2ml of blood samples were collected from peripheral vein by aseptic technique. The vein puncture site was cleansed with 70% isopropyl alcohol and 2% tincture iodine before collecting. The collected blood samples were inoculated into Tryptone soya broth and transported to the microbiology laboratory within 5–10 min at room temperature. The blood samples were collected prior to antibiotic treatment.

### Laboratory methods

#### Blood culture and bacterial identification

The blood culture bottles were incubated at 35–37 °C under aerobic conditions for 24 h and inspected daily for 7 days until growth detected. Bottles were observed macroscopically daily for visible evidence of bacterial growth such as hemolysis, turbidity, and gas production. Sub-culture was done on MacConkey agar and incubated aerobically at 35–37 °C for 24 h. Whereas, sub-culture was done on sheep blood agar and chocolate agar and incubated at 5% CO_2_ for 48–72 h. The same procedure was repeated until the seventh day before the blood cultures considered to be negative.

Bacterial identification was done through colony morphology, Gram stain and biochemical tests. Biochemical tests were performed on sub cultured media by isolating the pure colonies. For gram-positive bacteria, catalase, coagulase, novobiocin and mannitol fermentation were performed. For gram-negative bacteria, indole, citrate utilization, triple sugar iron, urea, mannitol, oxidase, nitrate reduction, lysine decarboxylase, lysine deaminase and motility test were carried out based on Clinical and Laboratory Standard Institute (CLSI) guideline 2020 [25].

#### Antimicrobial susceptibility testing

Antimicrobial susceptibility test was performed for each bacterial isolate based on Clinical and Laboratory Standard Institute guideline by using disc diffusion method on Muller Hinton agar [25]. Muller Hinton agar with 5% sheep blood was used for fastidious organisms. Approximately, three to five pure colony of the test organism was taken by using a sterile wire loop and emulsify in 2 ml of normal saline. McFarland (0.5) was used as a standard to check the 5% turbidity of bacterial suspensions. Then a sterile cotton swab was dipped in to the suspension and squeeze free from excess fluid against the inside wall of the test tube. The test organisms were uniformly seed on the surface of Muller-Hinton agar for non-fastidious group and Muller Hinton agar with 5% sheep blood for fastidious group and expose to a concentration gradient of antibiotic diffusing from antibiotic impregnated paper disc into the agar medium and the medium was incubated at 37 °C for 24 h.

Antimicrobial agents tested include erythromycin (15 µg), clindamycin (2 µg), chloramphenicol (30 µg), vancomycin (30 µg), trimethoprim/sulfamethoxazole (1.25/23.75 µg), ampicillin (10 µg), cloxacillin (30 µg), gentamicin (10 µg), amikacin (30 µg), ciprofloxacin (5 µg), ceftazidime (30 µg), ceftriaxone (30 µg) and cefotaxime (30 µg). Grades of susceptibility pattern were interpreted by comparison of the zone of inhibition according to CLSI guideline 2020 [25].

#### Quality control

Quality control of the culture media was performed for new batch of media. Visual inspection was done to check cracks in media, hemolysis, evidence of freezing and presence of air bubbles. The sterility of culture media was ensured by incubating 5% of each batch of the prepared media at 37 °C for 24 h. All prepared culture media were also checked by inoculating known strains such as *E. coli* (ATCC 25922) gram-negative bacteria, *S. aureus* (ATCC 25923) for gram-positive bacteria and *N. gonorrhoeae* (ATCC49226) for fastidious bacteria. To standardize the inoculums density of bacterial suspension for the susceptibility test, 0.5 McFarland standards was used.

### Statistical analysis

All data were encoded in Microsoft Excel 2016 and exported to Statistical Package for Social Sciences (SPSS) version 20 software. We used descriptive statistics, such as frequencies and percentages to describe the proportion of bacterial isolates and associated risk factors of neonatal sepsis. Binary logistic regression model was performed to determine the association between neonatal sepsis and associated factors. Moreover, all variables with a p-value < 0.25 in the bivariable analysis were included in multivariable analysis to account for possible confounding variables. P-value less than 0.05 considered as statistically significant. The strength of the association was interpreted using an odds ratio in a 95% confidence interval. Finally, the results presented on words, graphs and tables.

### Operational definition

MDR: MDR was defined as non-susceptibility to at least one agent in three or more antimicrobial categories.

Early onset sepsis: The onset of signs and symptoms of sepsis in neonates less than or equal to 3 days of life.

Late onset sepsis: The onset of signs and symptoms of sepsis after 3 days and up to 28 days of life.

Term: Babies born between 37 and 41 weeks of pregnancy.

Preterm: Babies born before 37 completed weeks of pregnancy.

Low birth weight: Newborn weight at birth < 2.5 kg.

Normal birth weight: Newborn weight at birth ≥ 2.5 kg.

## Results

### Socio-demographic characteristics of the study participants

Out of 400 sepsis suspected neonates, 246 (61.5%) were males. About 166 (41.5%) of the neonates had low birth weight (< 2.5 kg), and 118 (29.5%) were preterm. The majority of neonates, 364 (91%) were in the first three days of life (Table 1).

**Table 1:** Socio-demographic characteristics of neonates suspected of sepsis at SPHMMC, Addis Ababa, Ethiopia from March to July 2020 (n = 400)

Variables	Categories	Frequency (%)
Gender	Male	246 (61.5)
	Female	154 (38.5)
Age category	≤ 3 day	364 (91%)
	>3–28 day	36 (9%)
Newborn term	Term	282 (70.5)
	Preterm	118 (29.5)
Birth weight (kg)	< 2.5	166 (41.5)
	≥ 2.5	234 (58.5)

### Proportion of bacterial isolates

**Fig. 1:**
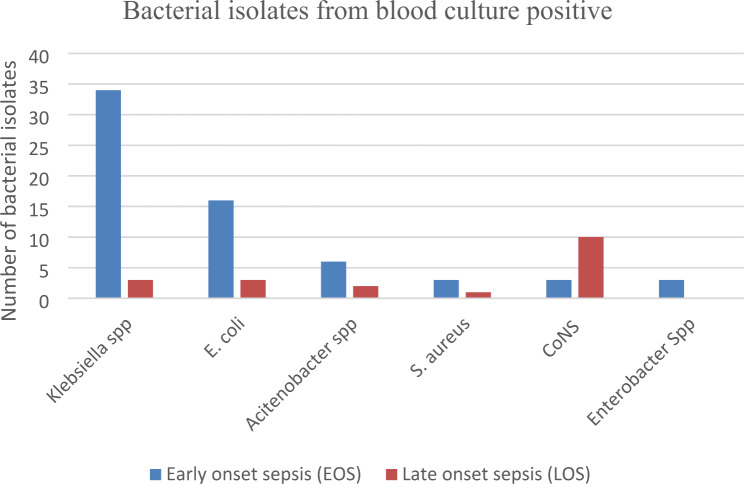
Distribution of bacterial isolates among neonates suspected of sepsis

Of all bacterial isolates, 67/84 (79.8%) were gram-negative bacteria, while the remaining 17 (20.23%) were gram-positive bacteria. The majority of culture confirmed cases of sepsis were in the first three days of life 65 (77.4%). About 59 (90.76%) of gram-negative bacteria were detected from the EOS, whereas 11 (57.87%) of gram-positive bacteria were detected from the LOS. The leading causes of neonatal sepsis in this study were *Klebsiella spp* 37 (44%) *and E. coli* 19 (22.6%) (Fig. 1).

### Antibiotic resistance pattern of bacterial species

*Coagulase negative staphylococcus* (*CoNS)* was found to be highly resistant to ampicillin 11 (85%), trimethoprim-sulfamethoxazole 10 (77%) and cloxacillin 13 (100%) (Table 2). The overall level of antibiotic resistance in gram-negative bacteria ranged from 13 (20%) to 54 (84%). Regarding specific bacterial species, *Klebsiella spp* were extremely resistant to gentamicin 35 (95%) and cefotaxime 35 (95%). Similarly, *Acinetobacter spp* showed the highest resistance to gentamycin 8 (100%) and trimethoprim-sulfamethoxazole 8 (100%). *E. coli* was resistant to ceftazidime and cefotaxime at the rate of 19 (100%) and 13 (68%) respectively. On the other hand, almost all identified gram-negative bacteria showed least resistance rate to ciprofloxacin and amikacin (Table 2).

**Table 2:** Antibiotic resistance pattern of bacterial species from neonates suspected for sepsis at SPHMMC, Addis Ababa, Ethiopia from March 2020 to July 2020 (n = 400)

Bacterial species	Bacterial resistance rate to antimicrobial agents (%)								
	AMP	AMK	GEN	CIP	CAF	SXT	CAZ	CTX	CLO
Gram-positive									
*CoNS* (13)	11(85)	NA	9(69)	3(23)	6(46)	10(77)	8(62)	NA	13(100)
*Gram-negative*									
*Klebsiella spp*(37)	NA	13(35)	35(95)	9(24)	20(54)	27(73)	25(68)	35(95)	NA
*E. coli* (19)	13(68)	4(21)	11(58)	5(26)	8(42)	10(53)	19(100)	13(68)	NA
*Acinetobacter (*8)	NA	2(25)	8(100)	2(25)	NA	8(100)	7(88)	6(75)	NA
Total (n = 64)	13(20)	19 (30)	54(84)	16(25)	28(44)	45 (70)	51 (80)	54(84)	

*CoNS* = Coagulase negative Staphylococcus, AMP = Ampicillin = AMK = Amikacin, GEN = Gentamycin, CIP = Ciprofloxacin, SXT = Trimethoprim/sulfamethoxazole, CAF = Chloramphenicol, CAZ = Ceftazidime, CTX = Cefotaxime, CLO = Cloxacillin

NA: not applicable

### Multidrug resistance pattern of bacterial isolates

About 84% of bacterial isolates were resistant to three or more classes of antimicrobial agents. Around 59 (70%) of bacterial isolates were resistant to three different categories of antimicrobial agents, while 10 (11.9%) were resistant to four different classes of antimicrobial agents. MDR isolates were found in 8 (100%) of *Acinetobacter spp* and 35 (95%) of *Klebsiella spp* (Table 3).

**Table 3:** Multidrug resistance pattern of bacterial isolates from sepsis suspected neonates at SPHMMC, Addis Ababa, Ethiopia, March 2020 to July 2020 (n = 400)

Bacterial isolates	Degree of resistance (%)					Total MDR isolates ≥ R3
	R0	R1	R2	R3	R4	
*E. coli* (n = 19)	0 (0)	1(5.26)	3 (15.79)	10(52.6)	3 (15.7)	13 (68)
*Klebsiella spp* (n = 37)	0 (0)	0 (0)	1(2.70)	30 (83.7)	5 (13.5)	35 (95)
*Acinetobacter spp* (n = 8*)*	0 (0)	0 (0)	0 (0)	6 (87.5)	1(12.5)	8 (100%)
*CoNS* (n = 13)	0 (0)	1(7.69)	3 (23.08)	8 (61.54)	1(7.6)	9 (69%)
Total (n = 77)	0 (0)	2 (2.38)	9 (10.71)	59 (70)	10 (11.9)	65 (84%)

R0- No antibiotic resistance, R1- Resistance to one category of antimicrobial agent, R2- Resistance to two different categories of antimicrobial agents, R3- Resistance to three different categories of antimicrobial agents, R4- ≥Resistance to four different categories of antimicrobial agents

### Factors associated with neonatal sepsis

Bivariable and multivariable regressions were used to assess the association between associated factors and neonatal sepsis. Low birth weight was found to be associated with neonatal septicemia (AOR = 45.90, 95% CI = 15.14-123.081, P = 0.002). In addition, gestational age less than 37 weeks and neonates whose mothers infected with HIV were showed a higher odd to develop sepsis (AOR = 18.2, 95% CI = 6.835–27.541, P = 0.004 and AOR = 5.30, 95% CI = 2.864–13.583, P = 0.003) respectively (Table 4).

**Table 4:** Crude and adjusted odds ratio for the association of factors and sepsis among neonates at SPHMMC, Addis Ababa, Ethiopia from March to July 2020 (n = 400)

Variables		Total	BG (n = 84)	NGB (n = 316)	COR	(95% CI)	P-value	AOR	95%CI	P-value
Age	≤ 3 days	364	79	285	1					
	> 3-28days	36	5	31	1.66	0.63–4.41	0.308			
Sex	Male	246	56	190	1.32	0.79–2.21	0.274			
	Female	154	28	126	1					
Medical device	No	4	2	2	1					
	Yes	396	82	314	0.26	0.04–1.88	0.183			
Birth Weight	< 2.5 kg	166	76	90	53.8	19.13-150.47	0.001	45.9	15.14-123.08	0.002
	≥ 2.5 kg	234	8	226	1					
Newborn term	Preterm	118	65	53	19.1	10.52-134.68	0.001	18.2	6.83–27.54	0.004
	Term	282	19	263	1					
Maternal HIV status	Negative	385	75	310	1					
	Positive	15	9	6	6.20	2.14–17.95	0.001	5.30	2.86–13.58	0.003
Maternal nutritional status	Malnutrition	14	6	8	2.96	0.99–8.78	0.050			
	Non-malnutrition	386	78	308	1					
Maternal chronic disease	Yes	172	40	132	1.26	0.78–2.05	0.336			
	No	228	44	184						
Outcome	Improved	372	76	296	0.64	0.27–1.51	0.311			
	Died	28	8	20	1					

COR = Crude odds ratio, AOR = Adjusted odds ratio, BG = Bacterial growth, NBG = No bacterial growth

## Discussion

In Ethiopia, one-third of the neonatal deaths are caused by sepsis [26]. The spectrum of bacteria that cause neonatal sepsis changes over time, which necessitates ongoing assessment and monitoring [8]. In this study, the prevalence of confirmed blood culture sepsis was 21%. Similar results were reported from Dire Dawa, Ethiopia 23% [27], Nigeria, 22.4% [28] and Ghana 21% [29]. However, it was lower than studies in Ethiopia (Addis Ababa 27% [30], Tigray 36.6% [31], Gondar 46.6% [32],) and other countries such as Zambia 33% [33], Egypt 40.7% [34] and India 26.2% [35]. The lower prevalence of sepsis in our finding might be due to similar signs and symptoms of septicemia with non-bacterial infections such as viruses and fungi that can cause a negative blood culture result [36]. Another reason could be due to differences in diagnostic method, sample size, study settings and study period.

*Klebsiella spp* 37 (44%) and *E. coli* 19 (22.6%) were the most prevalent bacteria in our finding. On the other hand, previous studies reported that *S. aureus* and *CoNS* were the primary organisms causing newborn sepsis [32, 37, 38]. The higher prevalence of *Klebsiella spp* in this study could be explained by the fact that *Klebsiella spp* are prevalent in the hospital environment and can cause infection at delivery or during their hospital stay [39, 40]. It can also be due to poor personal hygiene of caregivers or families, as well as contaminated medical devices [41].

The current study has shown that EOS was more prevalent than LOS, with a proportion of 77.8% and 22.61%, respectively. The results were comparable to previous studies in Wolega, Ethiopia 75.5% [42], Myanmar 77.8% [43], and Nepal 78.3% [44]. This higher prevalence of EOS could be attributed to prematurity, low birth weight, and poor hygienic conditions during delivery [45]. In our finding, *Klebsiella spp* and *E. coli* were the primary cause of EOS while *CoNS* and *S. aureus* were the predominant cause of LOS. The findings were consistent with a study in Nepal [46] and South Africa [47]. However, African reports indicated that *S. aureus* and *CoNS* were the predominant causal agents of EOS [48]. These inconsistent results might be due to the etiological agents of EOS and LOS change with time and geographical location [11]. Another reason could be due to early horizontal transmission in the delivery room or vertical transmission from maternal genital tract colonized with these pathogen [41, 49, 50].

The trend of antibiotic resistance change over time and may vary among countries [51]. Many literatures recommend ampicillin and gentamicin as a first-line treatment for neonatal sepsis [51–53]. Conversely, our finding showed that the majority of bacterial isolates were resistant to ampicillin and gentamicin, ranging from 68 to 85% and 75–100% respectively. This was consistent with previous studies in Ethiopia that showed high resistance rates of bacteria against ampicillin and gentamicin (90–100%) [31, 54]. Similarly, almost all bacterial isolates were 100% resistant to ampicillin in Egypt [55] and India [38]. The reason for this high resistance may be related with frequent use of these antibiotics.

In our study, the number of *S. aureus* and *Enterobacter species* isolates were very low to discuss about their susceptibility pattern, so we have discussed about bacterial species that have sufficient number. Accordingly, *CoNS* were the only gram-positive bacteria that have adequate number and showed least sensitivity to gentamycin (69%), ampicillin (85%) and cloxacillin (94%) respectively. This finding agreed with previous studies from Tanzania [56] and Nepal [57] that reported the resistance rate of *CoNS* to gentamicin (75%) and ampicillin (100%). The reason for this high resistance may be related with frequent use of these antibiotics which create selective pressure of antimicrobial agents.

Amikacin and ciprofloxacin were effective against *E. coli* and *Klebsiella spp*, which was comparable to previous study [54]. This can indicate that these two antibiotics could be a potential choice for empiric treatment of neonatal sepsis in the future.

The prevalence of MDR was 84% in our study, which was slightly lower than a previous study in the same setting, where the rate was 88% [58]. However, it was higher than other studies [24, 38, 44, 59]. The proportion of MDR among gram-negative bacteria was 87%, which was consistent with previous studies in Bahir Dar, Ethiopia with 88.2% [60], Ghna 71.7% [61] and Nepal with 81% [44]. The high percentage of MDR in gram-negative bacteria may be due to their ability to transfer genes, have intrinsic antibiotic resistance and produce beta-lactamase.

We also assessed associated factors of neonatal sepsis. Septicemia neonates with low birth weight were 45.90 times more likely to develop sepsis than neonates with normal birth (AOR = 45.9, 95% CI = 15.14-123.08, P = 0.002). This finding was consistent with a study in Gondar and Bahir Dar, Ethiopia [32, 60]. The significant association might be due to low birth weight newborns are mostly premature, underdeveloped immune system, unable to feed, and easily lose their heat which may increase the likelihood of neonatal infections. Preterm neonates with septicemia were 18.2 times more likely to develop sepsis than the term neonates (AOR = 18.2,95% CI = 6.83–27.54, P = 0.004), this was similar to previous studies [32, 62]. This association could be due to weaker immune system of the preterm infants that can easily develop systemic infection [63]. This can be better explained by the fact that neonates have low neutrophil storage pools which increases the occurrence of reduced number of neutrophils with serious infection [64]. Neonates whose mothers were seropositive were five times more likely to develop sepsis (AOR = 5.30, 95% CI = 2.86–13.58, P = 0.003). This might be due to the fact that mothers who are infected with HIV have low immune status, which causes infants to be susceptible to infection.

Age, sex, and maternal nutrition status were not significantly associated with neonatal sepsis. In this study, the death rate due to sepsis was7% and was not significantly related to blood culture results. This result was much smaller than study which reported a 20.9% death rate [65], but higher than studies in Bahir Dar and Dessie, Ethiopia that reported 4.0% and 4.8% death rate [66, 67].

### Limitations

This study has several limitations: The first limitation was unable to perform drug resistance enzymes, cefoxitin and genes because of budget and facility limitations. Laboratory analysis was the second limitation which fail to detect some pathogenic bacteria such as Pneumococcus and Group B streptococcus. Another limitation was data collection tool (questionnaire) for maternal related factors which suffers from recall bias. Therefore, care must be taken in interpreting and drawing conclusions based on such information, as there is a tendency for respondents to provide what they believe. The result from this study could not infer to the general population because of the nature of the cross-sectional study at a single center.

## Conclusions

In conclusion, EOS was caused primarily by *Klebsiella spp* and *E. coli*, while LOS was caused by *CoNS*. Gram-negative bacteria were highly resistant to gentamycin, ceftazidime and cefotaxime. Similarly, *CoNS* was also resistant against gentamycin, ceftazidime and cloxacillin. In addition, low birth weight, gestational age less than or equal to 37 weeks and maternal HIV status were associated with neonatal sepsis. Therefore, periodic review of antibiotic resistance profile is recommended. Further, large scale study is important to characterize each bacterial strain and to study more risk factors.

## Data Availability

The datasets used and/or analyzed during the current study are available from the corresponding author on reasonable request.

## Abbreviations

*CoNS*: Coagulase negative Staphylococcus
EDHS: Ethiopian demography and health survey
EOS: Early onset sepsis
GBs: *Group B streptococcus*
LOS: Late onset sepsis
MDR: Multidrug resistance
NICU: Neonatal intensive care unit
